# Predicting the risk of amputation and death in patients with diabetic foot ulcer. A long‐term prospective cohort study of patients in Tanzania

**DOI:** 10.1002/edm2.336

**Published:** 2022-04-06

**Authors:** Zulfiqarali G. Abbas, Nachiappan Chockalingam, Janet K. Lutale, Roozbeh Naemi

**Affiliations:** ^1^ 92976 Muhimbili University of Health and Allied Sciences Dar es Salaam Tanzania; ^2^ Abbas Medical Centre Dar es Salaam Tanzania; ^3^ Centre for Biomechancis and Rehabilitation Technologies, School of Health, Science and Wellbeing Science Centre Staffordshire University Stoke on Trent UK

**Keywords:** Africa, amputation, foot ulcer, mortality, peripheral arterial disease, peripheral neuropathy

## Abstract

**Introduction:**

This prospective cohort study aimed to identify the characteristics of patients with diabetic foot ulcer who are at higher risk of amputation and at increased risk of death.

**Methods:**

About 103(M/F:60/43) participants, with active foot ulcer at baseline, participated in this study and followed for 22 years till death or lost to follow‐up. Ten clinical measures were collected at baseline. During the follow‐up of 4.2 ± 5.4 years, 22(M/F:14/8) participants had an amputation and 50(M/F:32/18) participants passed away during 5.5 ± 5.8 years follow‐up period.

**Results:**

Cox Proportional Hazard regression (HR[95%CI]) indicated neuropathy (6.415[1.119–36.778]); peripheral arterial disease (PAD) (9.741[1.932– 49.109]); current smoking (16.148[1.658–157.308]); diabetes type‐ 1 (3.228[1.151–9.048]) and longer delay attending appointment after ulcer (1.013[1.003–1.023]) were significantly (*p* < .05) associated with increased risk of amputation. In addition, death was significantly associated with the risk of amputation (3.458[1.243–9.621]). Three parameters (HR[95%CI]) including neuropathy (3.058[1.297–7.210]); PAD (5.069[2.113–12.160]); amputation history (3.689[1.306–10.423]) and retinopathy (2.389[1.227–4.653]) were all significantly associated with increased risk of death. Kaplan–Meier survival analyses indicates that the time to amputation in years for participants who eventually died was significantly shorter (11.122 ± 1.507) vs those who stayed alive (15.427 ± 1.370).

**Conclusion:**

Neuropathy and PAD were the only two characteristics that increased both the risk of amputation and death. Amputation showed to contribute to an increased risk of death and those participants who eventually died had a higher risk of amputation. Delay in attending appointments after ulceration is shown to increase the risk of amputation. In addition, the participants with PAD showed a significantly shorter time to both amputation and death while neuropathy was only associated with decreased time to death. Amputation history and death during follow‐up decrease the time to death and amputation respectively.

## Graphical abstract

Neuropathy and PAD were the only two characteristics that increased both the risk of amputation and death. Amputation showed to contribute to increased risk of death and those patients who eventually died had higher risk of amputation. This two‐way reciprocal association between risk of amputation and death indicates the closely interlinked relationship between morbidity and mortality in patients with active diabetic foot ulcer. Cumulative hazrd of death is presented as a function of follow‐up duration in years in the image.

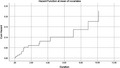

**Novelty statement**

What is already known?

- Amputation risk factors were identified as male sex, smoking history, ulcer history and body mass index, while risk factors for death were age, male sex, PAD and renal disease.

What this study has found?

- Neuropathy and PAD were the only two characteristics that increase both the risk of amputation and death, while diabetes type‐1 and retinopathy were associated with amputation and death respectively.

What are the implications of the study?

- In African patients with DFU, in addition to PAD and neuropathy, diabetes type and retinopathy should be considered to assess the long‐term risk of amputation and death.

## 1 INTRODUCTION

Diabetes is a leading cause of morbidity and mortality in both developed and developing world and impose a burden on the health sector^1^, ^2^, ^3^, ^4^

Diabetic foot ulcers (DFU) are associated with the highest morbidity and mortality.^1^, ^2^, ^3^, ^4^, ^5^, ^6^, ^7^, ^8^ Diabetic foot complications are the main cause of non‐traumatic lower‐limb amputation with rates of about 40%–60%. About 85% of these amputations are preceded by ulceration.^1^, ^2^, ^4^, ^9^, ^10^, ^11^ About 25% of those with diabetes will be affected by a foot ulcer during their life time.^9^, ^12^ With one lower limb amputation, people with diabetes have a 50% risk of getting a serious lesion in a second limb within two years and have 70% rate of death in five years following the initial amputation.^1^, ^2^, ^3^, ^4^, ^9^, ^10^, ^11^ The mortality risk rate at ten years for people with DFU is twice as compared with those who have no DFU.^13^

Diabetes imposes a heavy burden on the health services in most African countries. We previously estimated the expenses to both the patient and society of treating DFU in five countries with widely varying health care practices, reimbursement policies and gross domestic products.^14^, ^15^ Infection and gangrene are the most common precursors of amputation and the prevalence of amputation seen across Africa are therefore very high.^1^, ^7^, ^16^

Mortality rates are also high in African patients with DFUs.^1^, ^7^, ^16^ A high mortality rate (29%) was reported in Tanzania by Abbas and colleagues among patients with foot ulcer and was significantly higher among patients with PAD, neuro‐ischaemia, late presentation or non‐healing ulcers.^7^ The mortality rate was 54% in those who presented with established gangrene.^7^ The highest mortality rate has been documented in those who decline amputation of the relevant limb.^7^ This imposes a heavy socioeconomic burden.^15^ The prevalence of diabetic foot complications in Tanzanian populations has been previously reported in detail.^1^, ^2^, ^3^, ^4^, ^6^, ^7^, ^8^, ^17^ In order to decrease the socioeconomic cost associated with diabetic foot complications, a knowledge of the risk factors for amputation and mortality following diabetic foot ulceration is necessary.

A systematic review of the literature and meta‐analysis identified the male sex, a smoking history, a history of foot ulcers, osteomyelitis, gangrene, a lower body mass index and a higher white blood cell count as a predictive risk factor for amputation.^18^ There has been a focus on short term mortality rates after ulceration that is, 40% at 5 years, with the common risk factors for death identified as age, male gender, PAD and renal disease in systematic review of literature.^19^

While there has been an abundance of studies on the short to medium‐term outcomes of diabetic foot ulceration with regards to amputation and death which have been highlighted in the systematic reviews of the literature,^18^, ^19^ only a few studies focused on the factors that can identify the long‐term (i.e., 10 years or more) outcome of diabetic foot ulcer in relation to amputation and death.^20^ In the medium to long‐term follow‐up studies age, being on dialysis, and PAD were reported as the significant predictors of amputation.^20^ Significant predictors for death were reported as age, male sex, chronic renal insufficiency, dialysis and PAD.^20^

The current study is a unique investigation conducted in Dar es salaam, Tanzania, East Africa, on African patients with follow‐up for 22 years (January 1998–December 2020). The purpose of our study was to prospectively look for the limb and person survival with DFU patients during a follow‐up period of more than two decades. The overall aim of this study was to identify the risk factors that are associated with the future amputation and death in patients with diabetic foot ulcers in Tanzania. The first objective of this study is to identify the characteristics that increase the risk (hazard) of amputation and death in this group of patients. The second objective of this study was to identify the characteristics of patients with (against patients without) amputation or death during the follow‐up.

## 2 METHODS

### 2.1 Participants

Participants were recruited from patients who attended the clinic with active foot ulcer between January, 1998, and December, 1999. All data were collected in a specialist clinic located within a city. The primary inclusion criteria were the patient should be diagnosed with diabetes and the presence of any DFU at baseline. DFU was defined as a full‐thickness wound involving the foot or the ankle, distal to and including the malleoli. A total of 123 participants were recruited during the study period. The patients (participants) recruited during this period of two years were followed up for more than two decades till 2020 (1998–2020). All participated were African in origin and during the follow up all necessary medical or surgical interventions that were necessary for the management of DFU were done during follow‐up period. Analysis was only feasible by following participants very closely to establish intervention and outcomes, until death. A total of 103 (M/F:60/43) participants were eligible for analysis with active foot ulcer at baseline till the outcome known at the end of 2020 for this study period.

### 2.2 Data collection

A set of 9 categorical and 5 continuous parameters were collected from the participants during a single visit at baseline.

#### 2.2.1 Categorical parameters

The general categorical parameters were as follows: gender, presence of retinopathy and diabetes type, smoking (Current smoker, Never smoked, and Previous smoker), previous amputation and history of ulceration, according to the protocols set by IWGDF.^21^ The foot‐specific categorical parameters included the following: neuropathy (using 10‐g monofilament loss of sensation was assessed on both feet at 10 sites including Hallux, 3^rd^ Toe, 5^th^ Toe, 1^st^ meta tarsal head (MTH), 3^rd^ MTH, 5^th^ MTH, lateral midfoot, medial midfoot, centre of the hindfoot and dorsum of the foot.^22^, ^23^ The presence of PAD, ulcer recurrence frequency were also considered to be present based on the protocol proposed in IWGFD guidelines.^24^

#### 2.2.2 Continuous parameters

The continuous parameters included were as follows: age (year), Body Mass Index (Kg/m^2^), fasting blood sugar (mmol/L), duration of diabetes (Years) and delay attending appointment after ulcer (days).

### 2.3 Follow‐up

The participants were followed until their first amputation or death or until censored (lost to follow‐up). During follow‐up of (Ave ± STDEV) 4.2 ± 5.4 years, 22 (M/F:14/8) participants had amputation. During follow‐up of (Ave ± STDEV) 5.5 ± 5.8 years, 50 (M/F:32/18) patients passed away. All participants were asked to follow the usual footcare that they were instructed by the diabetic foot nurse when they attended their appointments. Tight control of diabetes, regular education almost once in every three months, any foot lesion even minor should immediately been reported, checking for cuts, blisters or cracks, applying moisturising cream and avoiding cutting nails—only done at the centre were adhered to during the follow‐up. All footwear were hand made by our local cobbler instructed by the clinical team to ensure proper offloading. The typical footwear we provided had a rigid sole, extra depth and width, rocker sole, shock absorbing insoles, waterproof, mostly Velcro fastening, but not slip‐on.

The management of risk factors (i.e., tobacco use, diabetes, low‐density lipoprotein levels and hypertension) was considered as standard therapy for all patients with PAD regardless of PAD classification. Therefore, concurrent therapy with the medical and revascularization strategies was considered, as whenever necessary antiplatelet agents and angiotensin‐converting enzyme (ACE) inhibitors were used. For the management of other risk factors such as tobacco use, low‐density lipoprotein levels and hypertension were prescribed. Glycaemic control was done on regular basis by taking fasting or random blood glucose on every follow‐up visit.

### 2.4 Data analyses

All statistical tests were performed using IBM^®^ SPSS^®^v.25.

#### 2.4.1 Assessment of the associations with the incident of amputation and death.

Cox Univariate Regression was utilized to assess the association of each of the categorical and continuous parameters with the risk of amputation and death (Hazard Ratio‐HR) during follow‐ip.

#### 2.4.2 Assessment of differences in the amputation and survival time for different sub‐groups.

In addition, Kaplan–Meier survival analyses were used to compare the differences in amputation free and survival times during follow‐up for categorical parameters.

#### 2.4.3 Assessment of differences in continuous parameters

Mann–Whitney U test was used to assess the differences in continuous parameters between the groups with vs without amputation and death during follow‐up.

## 3 RESULTS

About 103(M/F: 60/43) participants, with active foot ulcer at baseline, participated in this study, and the data were collected at the base line. During the follow‐up of (Ave ± STDEV) 4.2 ± 5.4 years, 22 (M/F:14/8) participants had an amputation and the remaining 81 were censored. The amputations were 12 minor and 10 major amputations.

In addition, during the follow‐up of (Ave ± STDEV) 5.5 ± 5.8 years, 50 (M/F:32/18) participants passed away and the remaining 53 were censored. From the 50 deaths, 25 were due to sepsis, 10 due to renal failure, 5 due to MI and 1 due to CCF, 4 due to aging and 5 others were unknown.

Tables 1 and 2 represent the results of categorical measures related to amputation and death respectively during the follow‐up. Tables 3 and 4 represent the results of the continuous parameters association with amputation and death respectively during the follow‐up.

**TABLE 1:** Associations between the Categorical parameters and the amputation incident

*Amputation*	All (103)		No amputation during follow‐up (80–77.7%)		With Amputation during follow‐up (19– 18.4%)		Survival Analysis Cox Univariate Regression		Kaplan–Meier survival Analyses	
Categorical Variable	Count	%	Count	%	Count	%	Hazard Ratio (95% CI)^a^	*p* Value^a^	Estimated Mean reduction in survival (years)^b^	*p* Value for reduction in survival^b^
Female	43	41.7	35	43.2	8	36.4	0.939(0.375–2.349)	0.892	−0.62	.892
Diabetes Type 1	13	12.6	8	9.9	5	22.7	3.228(1.151−9.048)	**0.026**	7.51	.**018**
Retinopathy	18	18.4	11	14.5	7	31.8	2.077(0.743–5.811)	0.164	3.03	.155
Neuropathy	84	81.6	66	81.5	18	81.8	6.415(1.119–36.778)	**0.037**	1.94	.431
Previous Ulceration	34	33	30	37	4	18.2	0.199(0.034–1.157)	0.072	.31	.180
Amputation history	8	7.8	4	4.9	4	18.2	2.265(0.225–22.753)	0.487	8.82	.180
Peripheral Vascular Disease	15	14.6	9	11.1	6	27.3	9.741(1.932–49.109)	**0.006**	8.97	.**003**
Wagner 1	1	1	0	0	1	4.5				
Wagner 2	63	61.2	55	67.9	8	36.4	0.016(0.001–0.252)	**0.003**		
Wagner 3	13	12.6	9	11.1	4	18.2	0.170(0.010–2.831)	0.217	4.74^c^	.**000**
Wagner 4	25	24.3	16	19.8	9	40.9	0.037(0.002–0.666)	**0.025**	10.53^c^	.**000**
Wagner 5	1	1	1	1.2	0	0	0.000(0.000–0.000)		.987	
Never smoked	84	81.6	67	82.7	17	77.3				
Past smoker	8	7.8	5	6.2	3	13.6	0.998(0.175–5.684)	0.999	8.3^d^	.216
Current smoke	11	10.7	9	11.1	2	9.1	16.148(1.658 −157.308)	**0.017**	.86^d^	.216
Recurrence	44	42.7	29	35.8	15	68.2	1.844(0.325–10.466)	0.490	2.81	.506
Died	50	48.5	34	42.0	16	72.7	3.458(1.243 −9.621)	**0.017**	4.31	.**011**

^a^ Based on univariate Cox Survival Analyses.

^b^ Based on Kaplan–Meier survival Analyses.

The *p* values less than .05 indicate a significant association and are shown in bold in the table.

^c^ In comparison to Wagner 2.

^d^ In comparison to Never smoked.

**TABLE 2:** Associations between the Categorical parameters and the death incident

*Death*	All (103)		No death during follow‐up (53–51.5%)		With death during follow‐up (49– 47.6%)		Survival Analysis Cox Univariate Regression		Kaplan–Meier survival Analyses	
Categorical Variable	Count	%	Count	%	Count	%	Hazard Ratio (95% CI)^a^	*p* Value^a^	Estimated Mean reduction in survival (years)^d^	*p* Value for reduction in survival^d^
Female	43	41.7	25	47.2	18	36.0	0.695(0.389–1.240)	0.218	−2.46	.216
Diabetes Type 1	13	12.6	7	13.2	6	12.0	0.845(0.357–1.997)	0.701	−.746	.700
Retinopathy	18	18.4	6	12.2	12	24.5	2.389(1.227–4.653)	**0.010**	4.99	.**008**
Neuropathy	84	81.6	42	79.2	42	84.0	3.058(1.297–7.210)	**0.011**	5.52	.**007**
Previous Ulceration	34	33.0	23	43.4	11	22.0	0.769 (0.320 – 1.849)	0.557	−.44	.**011**
Amputation history	8	7.8	0	0.0	8	16.0	3.689(1.306 – 10.423)	**0.014**	5.46	.**011**
Peripheral Vascular Disease	15	14.6	2	3.8	13	26.0	5.069(2.113–12.160)	**0.000**	7.40	.**000**
Wagner 1	1	1.0	0	0.0	1	2.0				
Wagner 2	63	61.2	33	62.3	30	60.0	0.450(0.051–3.963)	0.472		
Wagner 3	13	12.6	8	15.1	5	10.0	0.615(0.058–6.563)	0.687	.54^c^	.354
Wagner 4	25	24.3	11	20.8	14	28.0	0.832(0.088–7.881)	0.872	2.24^c^	
Wagner 5	1	1.0	1	1.9	0	0.0	0.000(0.000–0.000)		.988	
Never smoked	84	81.6	43	81.1	41	82.0		0.617		.690
Past smoker	8	7.8	3	5.7	5	10.0	0.575(0.190 – 1.736)	0.326	1.40^d^	.690
Current smoke	11	10.7	7	13.2	4	8.0	0.965(0.285 – 3.266)	0.954	3.43^d^	.690
Recurrence	44	42.7	16	30.2	28	56.0	1.628(0.725–3.656)	0.237	1.25	.867
Amputated	22	21.4	6	11.3	16	32.0	1.244(0.686–2.258)	0.472	1.47	.471

^a^ Based on univariate Cox Survival Analyses.

^b^ Based on Kaplan–Meier survival Analyses.

The *p* values less than .05 indicate a significant association and are shown in bold in the table.

^c^ In comparison to Wagner 2.

^d^ In comparison to never smoked.

**TABLE 3:** shows the continuous parameters for all participants and for the groups with and with no amputation during follow‐up

***Amputation***	**All**			**No Amputation during follow‐up**			**With Amputation Occurred during follow‐up**			**Survival Analysis Cox Univariate Regression**		**Mann‐Whitney *U* test**		
Continuous Parameter	Mean	Stdev	*N*	Mean	Stdev	*N*	Mean	Stdev	*N*	HR (CI at 95%)^a^	*p*‐ Survival^a^	*p*‐Difference^b^	Effect Size^b^	Effect Size Category^b^
Age (year)	52.4	12.8	103	52.1	12.2	81	53.3	15.1	22	1.003(0.971–1.037)	.841	.673	0.041	small
Body Mass Index (Kg/m^2^)	25.8	5.2	85	25.9	5.4	65	25.5	4.4	20	0.955(0.869–1.049)	.337	.848	0.020	small
Fasting Blood Sugar (mmol/L)	13.3	6.4	103	13.5	6.5	81	12.6	6.21	22	1.004 (0.933–1.080)	.925	.635	0.046	small
Duration of Diabetes (Years)	7.0	6.4	103	6.4	6.1	81	9.2	7.3	22	1.033(0.969–1.101)	.326	.105	0.159	Small/medium
Delay attending appointment after ulcer (days)	21.4	34.0	103	16.3	23.0	81	40.3	55.9	22	1.013(1.003 – 1.023)	.**010**	.**002**	**0.304**	Medium

Note

The *p* values less than .05 indicate a significant association and are shown in bold in the table.

^a^ Based on univariate Cox Survival Analyses.

^b^ Mann–Whitney *U* test.

**TABLE 4:** Continuous parameters for all participants and for the groups with and with no death during follow‐up

*Death*	All			No Death during follow‐up			With Death during follow‐up			Survival Analysis Cox Univariate Regression		Mann‐Whitney *U* test		
Continuous Parameter	Mean	Stdev	*N*	Mean	Stdev	*N*	Mean	Stdev	*N*	HR (CI at 95%)^a^	*p*‐ Survival^a^	*p*‐Difference^b^	Effect Size^b^	Effect Size Category^b^
Age (year)	52.4	12.8	103	49.9	12.1	53	55.1	13.2	50	1.030(1.007–1.054)	.**009**	.030	0.214	Medium/Large
Body Mass Index (Kg/m^2^)	25.8	5.2	85	26.1	5.1	47	25.6	5.4	38	0.943(0.885–1.006)	.075	.639	0.050	Small
Fasting Blood Sugar (mmol/L)	13.3	6.4	103	13.4	6.8	53	13.2	6.1	50	1.025(0.980–1.072)	.288	.955	0.005	Small
Duration of Diabetes (Years)	7.0	6.4	103	6.0	5.2	53	8.1	7.4	50	1.038(0.998–1.079)	.063	.254	0.112	Medium
Delay attending appointment after ulcer (days)	21.4	34.0	103	18.3	24.7	53	24.7	41.7	50	1.003(0.996–1.009)	.390	.665	0.042	Small

Note

The *p* values less than .05 indicate a significant association and are shown in bold in the table.

^a^ Based on univariate Cox Survival Analyses.

^b^ Mann–Whitney *U* test.

**FIGURE 1:**
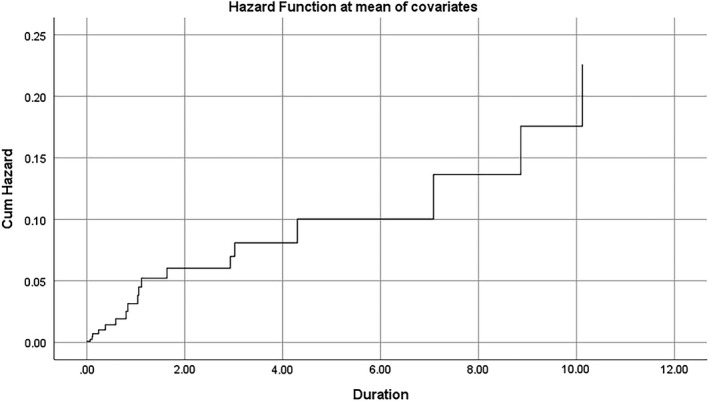
Total risk (cumulative hazard) for Amputation incident against the follow‐up duration in years

**FIGURE 2:**
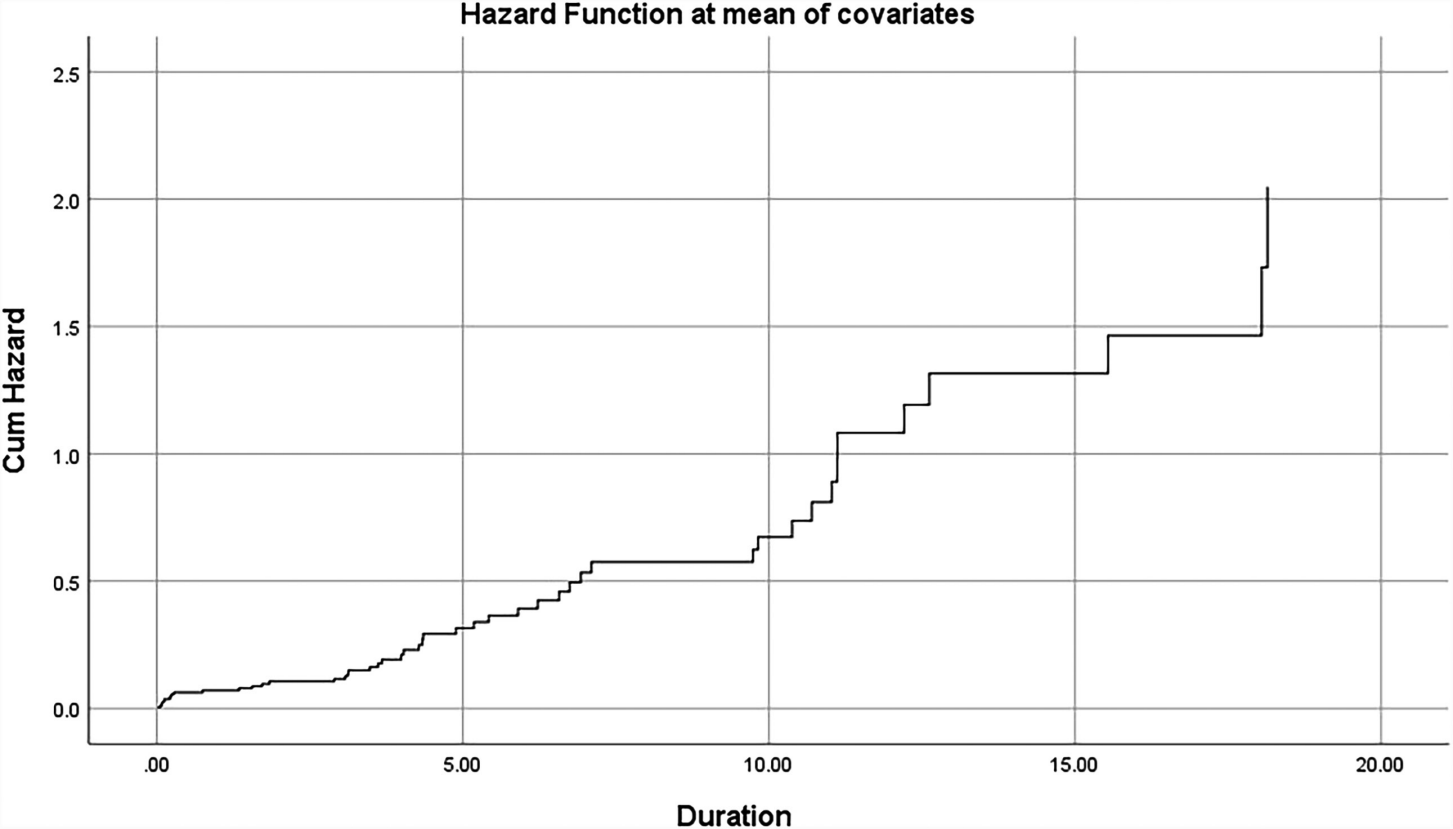
Total risk (cumulative hazard) for death against the follow‐up duration in years

Figures 1 and 2 also show the cumulative hazard (total risk) for amputation and death against follow‐up duration in years.

### 3.1 Amputation

#### 3.1.1 Associations

A number of parameters (HR [95%CI]) including neuropathy (6.415 [1.119–36.778]); PAD (9.741 [1.932–49.109]); current smoking (16.148 [1.658–157.308]) and diabetes type‐1 (3.228[1.151–9.048]) were all significantly associated with increased risk of amputation. However, Wagner Degree 2 (0.016 [0.001–0.252]) compared to Wanger Degree 4 (0.037[0.002–0.666] was associated with a decreased risk of amputation. From the continuous parameters, delay in attending appointments after ulcer (in days) was the only factor that was significantly associated with the increased risk of amputation (1.013[1.003–1.023]).

#### 3.1.2 Differences

In addition, the time to amputation in years (Mean ± Std. Error) for participants with the following characteristics was significantly shorter: Type 1 diabetes (6.743 ± 3.162) vs type 2 diabetes (14.251 ± 1.097); with PAD (5.529 ± 1.888) vs. No PAD (14.499 ± 1.047); Wagner Degree 4 (5.390 ± 1.204) vs Wagner Degree 3 (11.177 ± 3.358) vs. Wagner Degree 2 (15.923 ± 1.014). The participants who passed away during follow‐up (died) had shown significantly shorter time to amputation (11.122 ± 1.507 years) vs those who stayed alive during follow‐up (15.427 ± 1.370 years). In addition, patients with amputation during the follow‐up showed to have significantly longer delay attending appointment after ulcer (40.3 ± 55.9 days) compared to their counterparts with no amputation (16.3 ± 23.0 days) with a medium effect size (*r* = .304).

### 3.2 Death

#### 3.2.1 Associations

A number of parameters (HR [95%CI]) including neuropathy (3.058[1.297–7.210]); PAD (5.069[2.113–12.160]); amputation history (3.689 [1.306–10.423]) and retinopathy (2.389[1.227–4.653]) were all significantly associated with increased risk of death. However, repeat ulcer frequency (0.808 [0.682−0.958]) was associated with a decrease in risk of death. From continuous parameter, age was the only factor that was significantly associated with the increased risk of death (1.030[1.007–1.054]) during follow‐up.

#### 3.2.2 Differences

The time to death or the survival time in years (Mean ± Std. Error) was significantly shorter for participants with the following characteristics: With retinopathy (5.142 ± 1.439) VS. no retinopathy (10.140 ± 0.931); with neuropathy (8.042 ± 0.881) vs. no‐neuropathy (13.560 ± 1.523); with PAD (3.234 ± 1.000) VS. No‐ PAD (10.623 ± 0.899); amputation history (4.369 ± 1.502) vs. ulcer history (10.277 ± 1.553) or no ulcer or amputation history (9.834 ± 1.070). In addition, participants who dies during follow‐up showed to have significantly older age (Mean ± Stdev) 55.1 ± 13.2 years old compared with their counterparts who did not die 49.9 ± 12.1 years old with a medium/small effect size (*r* = .214).

## 4 DISCUSSION

### 4.1 **Associations with the risk of amputation and Differences in time to amputation**.

#### 4.1.1 Association with the risk of amputation

A number of parameters (HR [95%CI]) were found to be associated with the risk of amputation. Neuropathy (6.415 [1.119–36.778]), as in our previous study,^25^ we found neuropathy as one of the main risk factor for ulceration, which is also associated with risk of amputation as was indicated in the systematic review of patients with end‐stage renal failure.^26^

PAD (9.741 [1.932–49.109]); that is in line with the previous study in which the participants with a history of PAD, were found to have 23.06 times the risk of amputation compared with those with no history of PAD when they followed for a median of 36 months.^27^

In this study, it was shown that the current smoking (HR:16.148) was associated with an increased risk of amputation that is in line with a previous meta‐analysis study.^18^ Although in that study only the Odds Ratio:1.19 was reported, and the more important aspect of long‐term follow‐up was not investigated.

Diabetes type‐1 (3.228[1.151–9.048]) was also significantly associated with an increased risk of amputation that is different from the previous finding based on the systematic review of the literature where diabetes type was not found to be associated with the risk of amputation.^18^

However, Wagner Degree 2 (0.016 [0.001–0.252]) vs Wanger Degree 4 (0.037[0.002–0.666] was associated with a decreased risk of amputation. This is in line with the results of the study in which the Odds Ratio of Wagner grade 3 and 4 were found to be 13.10 times higher compared with those participants with an ulcer at Wagner grades 1 and 2.^7^, ^28^

#### 4.1.2 Differences in time to amputation

The time to amputation in years (Mean ± Std. Error) for participants with the following characteristics were significantly shorter: Type 1 diabetes (6.743 ± 3.162) vs type 2 diabetes (14.251 ± 1.097); with PVD (5.529 ± 1.888) vs. no PVD (14.499 ± 1.047); that is in line with the previous study where the presence of PAD was associated with an increased risk of death by 23.06 times.^27^ The time to amputation in years was significantly shorter for participants with ulcer with Wagner Degree 4 (5.390 ± 1.204) and Wagner Degree 3 (11.177 ± 3.358) vs. Wagner Degree 2 (15.923 ± 1.014). The participants who eventually passed away (died) had shown a significantly shorter time to amputation (11.122 ± 1.507) vs those who stayed alive (15.427 ± 1.370 ). The present study indicates that the participants with future amputation occurrence had distinctive characteristics in a set of parameters that were considered in the present study.

### 4.2 Associations with the risk of death and Differences in time to death

#### 4.2.1 Association with the risk of death

A number of parameters (HR [95%CI]) including neuropathy (3.058[1.297–7.210]) was found to be associated with increased risk of death. This is in line with the results reported in a previous study where altered sensation to monofilament was reported to increase the risk of death by 1.30 times.^27^ PAD (5.069[2.113–12.160]) was also associated with increased risk of death; that is in line with the previous study in which the participants with a history of PAD, were found to have an increased risk of death by 3.69 times when they followed for a median of 36 months.^27^ Amputation history (3.689 [1.306–10.423]); that seems to be contrary to the previous study in which the risk of death was reported to decrease in those with amputation history, that was reported as OR: 0.72.^27^ However, our results are in line with a previous study where lower extremity amputation was associated with the risk of mortality with a Hazard Ratio of 1.9–4.1.^29^ Retinopathy (2.389[1.227–4.653]) was also significantly associated with an increased risk of death in our study that is in line with the previous findings based on the systematic review of the literature.^30^ However, repeat ulcer frequency (0.808 [0.682 −0.958]) was associated with a decrease in risk of death.

#### 4.2.2 Differences in time to death

In addition, time to death or the survival time in years (Mean ± Std. Error) was significantly shorter for participants with certain characteristics.

The results of this study indicated that the participants with retinopathy (5.142 ± 1.439) VS. no retinopathy (10.140 ± 0.931) had a significantly shorter survival time; is in line with the previous study where the presence of retinopathy was associated with an increased risk of death by 1.08 times.^27^

In this study, we also found that participants with neuropathy (8.042 ± 0.881) vs. no‐neuropathy (13.560 ± 1.523) had significantly shorter survival time; that is in line with the previous study where the presence of neuropathy was associated with an increased risk of death by 1.30 times.^27^

The results also indicated that participants with PAD (3.234 ± 1.000) VS. those with No‐PAD (10.623 ± 0.899) showed to have significantly shorter survival time; that is in line with the previous study where the presence of retinopathy was associated with an increased risk of death by 3.69 times.^27^

In addition, in this study, we found amputation history (4.369 ± 1.502) vs. ulcer history (10.277 ± 1.553) or no ulcer or amputation history (9.834 ± 1.070); is in line with the previous systematic review where amputation was associated with increased risk of mortality.^30^

### 4.3 Strength and limitations

The present study is unique as it reports on a cohort of patients who were followed for a very long period (January, 1998–December, 2020) to identify the risk factors for future amputation and death. While different parameters seem to have been associated with the risk of amputation and death, PAD and neuropathy seem to be the common characteristic of patients with amputation or death when long terms complications of diabetic foot ulcers are investigated. While the number of participants in this study was limited, which makes it difficult to generalize these outcomes, it can be argued that this study is a steppingstone towards bigger studies.

### 4.4 Clinical implications and future directions for healthcare guidelines and policies

The results of this study indicated that both PAD and neuropathy were significantly associated with the risk of lower extremity amputation and death. Smoking has contributed to increased risk of amputation and amputation history has contributed to increased risk of death. It is also interesting to note that the higher the Wagner Degree classification, the shorter the time to amputation, which can have implications in stratifying patients with active diabetic foot ulcer.

In addition, the results indicated that the patients who passed away (died) during the follow‐up had shown significantly shorter time to amputation vs those who stayed alive. This needs to be considered in conjunction with the finding that time to death was significantly shorter for those with amputation history. The above indicates that a decrease in mortality associated with diabetic foot disease requires a significant reduction in amputations with implications in setting healthcare guidelines and policies. These can indicate the close interrelationship between amputation and death and can play a role in decreasing the morbidity and mortality associated with a diabetic foot ulcer. The longer delay attending appointment after ulcer is an important factor that need to be taken into account as it showed to have increased the risk of amputation. Hence, in clinical practice, further emphasis needs to be put on the immediate attendance of patients to the appointment as soon as an ulcer occurs.

As indicated before, since diabetes imposes a heavy burden on the health services in most African countries, the findings of this study have major implications in developing policies. In light of stratifying patients based on the findings of this study, the expenses for treating DFU to both the patient and society can be decreased. The outcome of this work has direct implications for health care practices, reimbursement policies and gross domestic products in Tanzania.

## 5 CONCLUSION

Participants who were vulnerable to future diabetic foot amputation and death during follow‐up have neuropathy and PAD. The results of the current study indicate a close interrelationship between amputation and death and associations between the two. It can be concluded that to achieve a reduction in mortality rates associated with diabetic foot disease, a significant reductions in amputations need to be achieved.

## **CONFLICT** OF **INTERESTS**

None declared.

## AUTHOR CONTRIBUTION

**Zulfiqarali G Abbas:** Data curation (lead); Funding acquisition (supporting); Investigation (lead). **Nachiappan Chockalingam:** Project administration (supporting); Writing – review & editing (supporting). **Janet K. Lutale:** Investigation (supporting). **Roozbeh Naemi:** Conceptualization (lead); Formal analysis (lead); Investigation (equal); Methodology (lead); Visualization (lead); Writing – original draft (lead); Writing – review & editing (lead).

## ETHICAL APPROVAL

This study used secondary anonymized data from a wider study conducted at Abbas Medical Centre, Dar es Salaam, Tanzania. All participants gave informed consent before taking part in the wider study.

## Data Availability

The data are available upon reasonable request.
